# Exam cheating among Quebec’s preservice teachers: the influencing factors

**DOI:** 10.1007/s40979-020-00062-6

**Published:** 2020-11-30

**Authors:** Sylvie Fontaine, Eric Frenette, Marie-Hélène Hébert

**Affiliations:** 1 Département des sciences de l’éducation, Université du Québec en Outaouais, Pavillon Alexandre-Taché, 283, boulevard Alexandre-Taché, Case postale 1250, succursale Hull, Gatineau, Québec, J8X 3X7 Canada; 2 Département des fondements et pratiques en éducation, Université Laval, Faculté des sciences de l’éducation, Pavillon des Sciences de l’éducation, local 366, 2320, rue des Bibliothèques, Québec (Québec), G1V 0A6 Canada; 3 TÉLUQ, Département Éducation, 455, rue du Parvis, Québec (Québec), G1K 9H6 Canada

**Keywords:** Cheating on exams, Preservice teachers, Academic integrity, Academic misconduct, Postsecondary education

## Abstract

This article presents the results of a research that aimed to examine the phenomenon of student cheating on exams in faculties of education in Quebec universities. A total of 573 preservice teachers completed an online survey in 2018. The questionnaire consisted of 28 questions with a Likert scale related to individual and contextual factors associated with the propensity to cheat on exams as well as two yes/no items on the arguments for cheating. Descriptive and hierarchical linear regression analyses highlighted the existence of cheating but also how three factors influenced the students’ propensity to cheat: influence of peers, methods of cheating, and institutional context.

## Introduction

Exams, tests, and essays are used in education systems worldwide to demonstrate students’ competencies, skills and knowledge (Fontaine et al. 2013; Stiggins 2009). Students’ academic results are an indication of their success and these results have social consequences: successful completion of a course, admission to a university program, winning a scholarship, earning a diploma and sometimes even finding employment. According to many authors, the pressure to succeed, using the metrics list above, can lead to cheating (Callahan 2004; Tchouata et al. 2014; McCabe et al. 2012; Lancaster and Clarke 2017). This phenomenon of cheating raises questions about the validity of grades and the credibility of diplomas awarded (Cizek 1999; Desalegn and Berhan 2014; Fendler et al. 2018). These validity and credibility questions are also relevant in the present context of COVID-19 pandemic which has suddenly imposed online teaching and assessments for teachers and students. To that effect, Corrigan-Gibbs et al., (Corrigan-Gibbs et al. 2015 p.28–29) point out that, with this new teaching and learning modality, “it will be crucial to preserve the same levels of trust, honesty, and integrity online that people expect from face-to-face interactions.”

Student cheating is not a new phenomenon. In fact, according to Fishman (2016), the first documented case of cheating, referring to the theft of an exam from a university printing office, has been reported in a research paper written by Barnes in 1904. Since then, the literature is replete with cases of cheating, albeit without a consensus about its prevalence. Some authors report that in general, as many as 80% of students are cheating (Qualls 2014) while others suggest numbers ranging from 30% to 60% (Bowers 1964; Jurdi et al. 2011; McCabe et al. 2001; Williams and Williams 2012). Statistics about cheating vary from one study to another, possibly because of differing definitions of cheating behaviors. For some authors, the statistics include cheating in written assignments (plagiarism) and on exams (Dodeen 2012) while others focus on cheating on exams only (Michaut 2013) or specifically, on plagiarism (Harper et al. 2019). And so, we observe a problem in the literature with the definitions of cheating that can include many or one type, the frequency of cheating and the generalization of statistics that refer to a mix of all these factors. Nonetheless, recent international research tend to confirm a high number of cases of cheating (Christensen Hughes and Mighty 2010; Ellahi et al. 2013; Fendler et al. 2018; Tchouata et al. 2014; Ma et al. 2008; McCabe et al. 2012; Stiles et al. 2017).

The phenomenon of cheating appears to be worldwide and at all levels from secondary school to university. Crittenden et al. (2009) studied the cheating culture within faculties of commerce in 36 countries. They define cheating culture as tolerance of cheating, beliefs in cheating and status of the cheating culture and targeted three specific predictors of cheating for their study: gender, level of corruption in the country, and socioeconomic environment. Their research showed that women are less likely to cheat than men, but also identified social factors that influence cheating, such as the level of corruption in the country and socioeconomic conditions. After illustrating the magnitude of the global phenomenon of cheating, they conclude that business students, who are future business leaders, appear to learn that *results* are more important than *learning*, and this, regardless of the way, ethical or not, they obtain these results (Crittenden et al. 2009). More recently, Miller et al. (2015) conducted a worldwide study of secondary school principals (35 countries) addressing cheating in secondary schools. They found that school principals within developing nations report more problematic cheating than school principals from wealthiest countries. They suggest that “schools in more economically disadvantaged nations may have more difficulty controlling undesired behaviors, such as cheating, among students” (p.226). Although there are few worldwide studies, large studies have been conducted in numerous countries (Michaut 2013; Harper et al. 2019; Denisova-Schmidt et al. 2019).

In Canada, Christensen Hughes and McCabe (2006) conducted a national study with 14,913 students from 11 universities that showed the prevalence of cheating, while indicating a higher proportion of self-reported cheating on exams (58%) and on written assignments (73%) in secondary school than at university level (respectively 18% and 53%). Although they did not survey secondary school directly, they asked first-year university students to reflect on their high school experience. Interestingly, their study also shows that students are sometimes confused about what constitutes serious cheating behavior. For example, collaborating with peers for a take-home exam is not perceived by students as serious cheating even though they know they are supposed to do it on their own as oppose to as a group. This confusion about the appropriateness, or not, of collaboration between students has been reported as well in Jurdi, Hage, and Chow’s study (Jurdi et al. 2011).

Exploring the literature on cheating on exams has led us to realize that while research in this area is abundant, there is a lack of Canadian research on this particular topic (Christensen Hughes and McCabe 2006; Jurdi et al. 2011; Wideman 2011) and most studies tend to simultaneously address both cheating on exams and plagiarism. Furthermore, few articles have reported on research addressing cheating on exams specifically by students in faculties of education (Bens 2010; Tchouata et al. 2014) and to our knowledge, no study has been conducted on this topic in a faculty of education of a Quebec university.

Among university students, those in the field of education will play a crucial role in the education of young people who will become the leaders of tomorrow. Preservice teachers in Quebec must develop 12 competencies, including competency number 12 “To demonstrate ethical and responsible professional behaviour in the performance of his or her duties” (Gouvernement du Québec 2001 p.55). Therefore, their teaching should reflect an ethical approach (Boon 2011; Jeffrey 2013; Jutras 2013) grounded on moral reasoning in their decision making (Cummings et al. 2007; Ndzedi 2016). These future teachers after graduation will assume a dual role as leaders in professional integrity and as models of integrity for their students (Boon 2011; Cummings et al. 2007). Taking stock of the phenomenon of cheating on exams in faculties of education thus becomes a preliminary yet essential step in any process aiming to ensure that future teachers are capable of assuming this dual role, especially in light of some research findings that demonstrate continuation of cheating in further studies and professional life (Christensen Hughes and McCabe 2006; Cronan et al. 2017; Ellahi et al. 2013; Novotney 2011).

### Research purpose and questions

We aim to further the research on individual and contextual factors associated with cheating among university students enrolled in education programs. A better understanding of these factors may allow university administrators and professors to focus on strategies to reduce cheating and “create ethical organizations” (Van Yperen et al. 2011 p.5).

More precisely, our research objective is to examine students’ propensity to cheat on exams in the faculties of education at five francophone universities in the province of Quebec, Canada. This objective brings us to five specific questions:

1. How big is the propensity to cheat on exams in faculties of education?
2. Why do preservice teachers decide to cheat on exams?
3. What methods are considered the best to cheat on exams?
4. What methods do students used to cheat?
5. What is the impact of specific individual factors, namely students’ academic goals, perception of control over tasks, engagement in studying, methods of cheating, and contextual factors, such as peers’ influence and institutional context, on the propensity to cheat?

### Theoretical framework

**Fig. 1:**
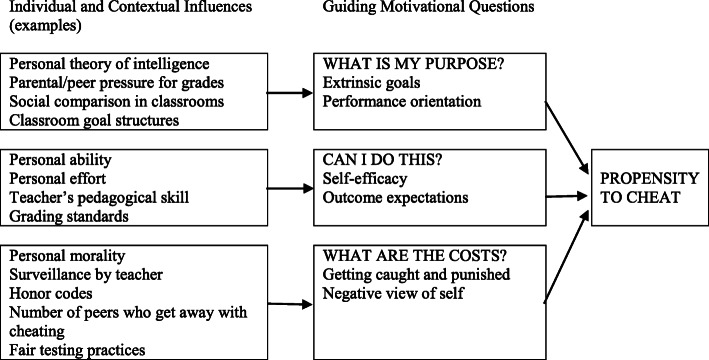
Proposed motivational framework for integration of the cheating literature (Murdock and Anderman 2006 p.130)

Murdock and Anderman (2006) propose a theoretical model of cheating that includes three components: students’ goals, their expectations regarding achieving these goals, and their evaluation of the cost to achieve these goals. As illustrated in Fig. 1, their model provides an excellent overview of all factors related to propensity to cheat in the literature.

In our research, we focused on seven factors included in this theoretical model: six independent factors and one dependent factor (propensity to cheat). Four of these factors are related to the individual, namely the type of academic goals students aim to achieve in their program of study, students’ engagement in their studies, choice of method of cheating (in order to explore students’ perception and use of various methods to cheat), and students’ perception of control over the tasks to be achieved. These factors fall under two questions articulated in Murdock and Anderman’s model, namely *what is my purpose?* and *can I do it?* We were also interested in two factors embedded in contextual influence – peers’ influence and institutional policy or code on integrity. These two factors fall under the question *What are the costs?* Murdock and Anderman (2006) demonstrate that when students focus on performance goals more than on mastery goals, when they have poor expectations of their abilities to achieve these goals despite their efforts, and when they “assess that potential costs incurred from cheating are minimal, they are more apt to engage in dishonest behaviors” (p.130). These factors have also been studied by numerous authors although not specifically in education faculties (Bernardi et al. 2008; Denisova-Schmidt et al. 2019; Ellahi et al. 2013; Meng et al. 2014; Rinn et al. 2014; Tas and Tekkaya 2010).

### Propensity to cheat on exams

It appears necessary to distinguish between cheating and plagiarism, two practices often studied together by researchers. Indeed, the literature on cheating usually includes cheating in written assignments, also called plagiarism, and cheating on exams. Plagiarism involves copying words or using a slightly modified text of an author without citation (Shei 2005; Walker 2010) in a written task where originality is expected (Fishman 2009). Cheating on exams, on the other hand, is a fraud committed by a student to increase the chances of success at examination (Chaput de Saintonge and Pavlovic 2004; Michaut 2013; Pavlin-Bernardić et al. 2017). This later definition was retained for the purpose of our study which focuses specifically on the propensity to cheat on exams.

### Students’ academic goals

Murdock and Anderman (2006) associate performance goals with extrinsic motivation, mainly externally influenced (school, peers), while mastery goals are intrinsic and influenced by personal goals. Both types of goals are academic goals. Performance goals reflect a “desire to demonstrate skills, either by trying to be better than others, or by trying to avoid being less good than others” (Tchouata et al. 2014 p.49). Students who pursue performance goals are more concerned with comparing themselves with others while students who pursue mastery goals are engaged in tasks, are concerned with self-improvement, and are eager to learn and to integrate new knowledge (Anderman and Danner 2008). Many researchers also conclude that pursuing performance goals may lead to cheating. For example, studies conducted by Anderman and Midgley (2004), Murdock et al. (2007), and Tas and Tekkaya (2010) conclude that having performance goals, or being in a class where grades are valued more than learning, is a predictor of cheating. Similarly, Tchouata et al. (2014), Olafson et al. (2013) concluded that when students pursue academic goals mainly for the purpose of achieving high grades or obtaining a diploma rather than for learning, cheating could become an option. Other research also states that “results generally indicate that personal mastery goals are inversely related to cheating, whereas personal extrinsic goals are related positively to cheating” (Anderman and Danner 2008 p.167). Recently, Anderman and Won (2019) conducted a study to explore the relation between academic motivation (mastery, extrinsic or avoidance goals), personality variables (impulsivity and sensation-seeking) and cheating. Contrary to previous studies, these researchers concluded that the type of goals was not predictive of cheating. However, it was predictive of the student’s belief about the *acceptability* of cheating. Therefore, pursuing extrinsic or avoidance goals were associated with the belief that cheating is an acceptable behavior.

### Students’ engagement in studying

Students’ engagement in their programs – as demonstrated by class attendance, amount of time spent studying, and procrastination in homework and study – have been studied as well (Ellahi et al. 2013; Guibert and Michaut 2009). Research has shown a link between a lack of engagement, manifested by poor class attendance and little time devoted to studying, and cheating (Ellahi et al. 2013), between partying and cheating (Whitley 1998), and between procrastinating and cheating (Patrzek et al. 2015). According to these researchers, students who are not very motivated by their studies, who devote little time to study and work, and who tend to procrastinate in their school tasks, are more likely to cheat. Students’ engagement might also be triggered by their perception of the relevance of what they learn. In her study, Bens (2010) suggests that students in teacher training programs may cheat when they do not see the applicability of the material they learn in classes. In other words, if it is not part of what they need in their real work as teachers, students may be more likely to cheat. Although these studies concur in associating the likelihood of cheating with a lack of engagement, others report that “surprisingly, students’ motivations toward reading, writing, and learning do not seem to have a valuable impact on the likelihood of their misconduct” (Grira and Jaeck 2019).

### Perception of control over tasks

Students’ perceptions of the degree of control they exercise over their school activity has also been studied by some researchers (Rettinger and Kramer 2009; Whitley 1998). The degree of control perceived by the student refers to the concept of “locus of control” associated with attribution theory (Weiner 1986). Despite the fact that the influence of the perception of one’s control over a task is not always clearly defined in the literature, researchers suggest some link between the perception of having little control over tasks and cheating (Rettinger and Kramer 2009; Whitley 1998). Similarly, Rinn et al. (2014) found a low to medium correlation between students’ perceptions of their ability to perform the task and a propensity to cheat.

### Methods of cheating

Cizek (1999) and Faucher and Caves (2009) propose the following three categories of methods to cheat. One category is using forbidden material during exams, for example notes on pieces of paper inside or outside the classroom or notes written on one’s own body. A second is taking, receiving or giving information about the exam that should not be shared. Examples of this second category include glancing at a peer’s copy of an exam, exchanging exams, and using sign language or codes for communicating answers. According to Bernardi et al. (2008), this latter method of cheating is facilitated when exams are composed of multiple-choice and true or false questions. Another method falling in this category is the reception by students of information about an exam from students of another cohort that has already written the same exam. By way of remedies, Christensen Hughes and McCabe (2006) suggest that “faculty may need to either substantially change their exams between semesters” or “for courses with multiple sections … create different exams or have all students write the same exam at the same time” (p.15). The third category of cheating methods is called “circumventing the process of assessment” (Faucher and Caves 2009 p.38). This category includes all excuses that students can provide to avoid the exam on the appointed day. Excuses such as sudden illness or the death of a grandmother (again!) are good examples. It is also worth noting that the development of technology opens the door to a wide range of new high-tech devices such as smartphones, smart watches, or earpieces can facilitate cheating on exams (Michaut 2013).

### Institutional context

Other reasons put forward for cheating are related to the institutional context. Most academic institutions have legal frameworks, policies, and regulations to guide practices related to student assessment. Whether it is a policy on academic integrity, a policy on plagiarism, or a code of honor, these documents are accessible to students and often reproduced, in part or in full, in the course outlines. While the literature clearly indicates a decrease in cheating associated with the existence of institutional policy on integrity or code of honor and cheating (McCabe et al. 2012; McCabe and Trevino 1993), it also stresses the importance of having consequences of cheating known and enforced in order to reduce cheating (Meng et al. 2014; Murdock and Anderman 2006; Schuhmann et al. 2013). In the context of online teaching, it seems that having a code of honor is far less effective than having stern warnings about the consequences of cheating while doing the exam (Corrigan-Gibbs et al. 2015). Yet, researchers who have studied the influence of these legal frameworks on the phenomenon of cheating conclude that, in many cases, students do not know about or understand these documents (Ellahi et al. 2013; Ma et al. 2008). They are unaware of the consequences associated with cheating and consider the risks to be low (Murdock and Anderman 2006; Ma et al. 2008; Meng et al. 2014; Schuhmann et al. 2013).

### Peers’ influence

As for institutional context, the behavior of peers is included in the question *What are the costs?* in Murdock and Adderman’s theoretical model (Murdock and Anderman 2006). The influence of peers on a student’s decision to cheat has been widely documented since the earliest studies on cheating (Bowers 1964; Crittenden et al. 2009; Cummings et al. 2002; Christensen Hughes and McCabe 2006; Ellahi et al. 2013; Kisamore et al. 2007; Ma et al. 2008; McCabe and Trevino 1997; Meng et al. 2014; Rettinger and Kramer 2009; Whitley 1998). Although there is consensus that peers’ influence exists, the extent of this influence is unclear. Some authors suggest that when students are competing against one another, thereby creating a competitive environment, it appears to lead to cheating (Cizek 1999; Whitley 1998). However, a directly competitive environment doesn’t seem to be necessary for students to cheat. In fact, mere knowledge that one’s peers are cheating would be sufficient to enhance the chance of a fellow student cheating as well. Students tend to “go with the flow” and justify their behavior by pointing out the commonness of the cheating practice (Crittenden et al. 2009; McCabe and Trevino 1997; Meng et al. 2014; Schuhmann et al. 2013). In that sense, Rettinger and Kramer (2009) argue that simply knowing that student friends are cheating, therefore being immersed in a “cheating culture”, and possibly leading to “peer pressure”, is enough to motivate a student to cheat. They argue that “knowing people who cheat (or have cheated) is a risk factor for starting to cheat” (p.296). From their study conducted with 600 university students in Ukraine, Denisova-Schmidt et al. (2019) report that the perception of the action of cheating, which they labeled “corruption”, from others, including peers in the classroom but also teachers, politicians, and relatives, would be sufficient for students to engage in cheating behaviors. In this respect, researchers (Meng et al. 2014; Pavlin-Bernardić et al. 2017; Smith et al. 2012) argue that the cheating student resorts to ‘neutralization’ techniques to justify his action (Sykes and Matza 1957). Thus, cheating students deny their responsibility and convince themselves that they do no harm to anyone, or that the victim (the teacher giving the exam) has deserved it or that everybody does it as well. These “neutralizing attitudes are positively correlated with student cheating” (Rettinger and Kramer 2009 p.295). Finally, the opinion of students and their entourage about the acceptability of cheating is linked to the decision to cheat (Meng et al. 2014; Crittenden et al. 2009; Schuhmann et al. 2013).

In summary, factors influencing students in their decision to cheat could be associated with each student and labeled “individual influences” (Schuhmann et al. 2013 p.9). They encompass students’ background characteristics, attitudes, perceptions, etc. (Yu et al. 2017). Other factors are related to the context (policy on integrity, code of honor, peers’ behaviors, etc.) and associated with the institution students are enrolled at (Christensen Hughes and Mighty 2010; Ellahi et al. 2013; McCabe et al. 2012).

## Method

### Measures

In this study, we used three sections of the *Cheating at the University Questionnaire* (CUQ); 1) demographic data, 2) the *University Cheating on Exams Questionnaire* (UCEQ) (Frenette et al. 2020), and 3) two yes/no items on the arguments for cheating. The other section, not used in this study, is related to perceptions of risk related to cheating. Demographic data are gender, age, year in the program and program. In order to answer research questions 1 (extent of the propensity to cheat), 4 (methods used to cheat) and 5 (impact of specific factors on the propensity to cheat), the 28-item UCEQ is used. Respondents were asked to rate each item using a 4-point scale which ranged from 1 = “strongly disagree”, indicating the absence of cheating, to 4 = “strongly agree” representing lots of cheating. Since the scale refers to the propensity to cheat on exams, a concept close to that of beliefs, attitudes and perception, the choice of a Likert scale was made. This questionnaire elicits preservice teachers’ self-ratings for the following seven factors: c*heating on exams* (*n =* 3; α = .77), *methods of cheating* (*n =* 3; α = .83), *institutional context* (*n =* 3; α = .54), *peers’ influence* (*n =* 3; α = .79), *perception of control* (*n =* 7; α = .72), *goal of performance* (*n =* 4; α = .64), and *engaged in studying* (*n =* 5; α = .61). Internal consistency for institutional context is considered low. Evidence of validity (Downing 2003) for the content (literature review, focus group, item generation, expert judgement, student judgement), response process (time of completion, response scale, and ethics), internal structure (pretest: interitem correlations, item-total correlations, internal consistency; data collection: interitem correlations, item-total correlations, internal consistency, and CFA^1^), relation to other variables (relation with the BIDR and gender differences) and consequential aspect were drawn from the data collected (Frenette et al. 2020). All these evidences support the use of the data obtained from this questionnaire.

For the purpose of exploring the preservice teachers’ propensity to cheat on exams (research question 2) and what methods are the best to do so (research question 3), two yes/no items on the arguments for cheating were used in which respondents indicate ‘yes’ or ‘no’ to a list of behaviors. Moreover, a short-form (13 items) of the *Impression Management* scale of the *Balanced Inventory of Desirable Responding* (BIDR) (D’Amours-Raymond 2011) was used to control social desirability bias since respondents may try to present themselves in a socially desirable way. Each statement was rated from 1 (false) to 7 (totally true) and dichotomized as 0 (score of 1 to 5) or 1 (score of 6 and 7) to identify extreme responses indicating social desirability (Paulhus 1984). Internal consistency of the *Impression Management* scale is considered low, but acceptable (Cronbach alpha = 0.60).

### Participants and procedures

In winter 2018, the link to the online survey was sent by email by the universities to a convenience sample of approximately 5500 preservice teachers enrolled in the faculties of education of five Quebec universities. A total of 573 students (486 females, 86 males, 1 other) completed the survey on LimeSurvey. The sample’s characteristics are presented in Table 1.

**Table 1:** Characteristics of respondents: age, year in program, and program

Age		Year in the program		Program	
18–20	17.80%	1st	27.92%	Kindergarten/primary program	47.47%
21–23	48.52%	2nd	24.26%	Secondary	17.45%
24–25	12.39%	3rd	23.91%	Special education	19.55%
26 and up	21.29%	4th	17.98%	Other (e.g. arts, physical activity)	15.53%
		Special case	5.93%		

### Analysis

Descriptive statistics (frequency counts and percentages) were employed to answer research questions 1 (extent of propensity to cheat), 2 (reasons for cheating), 3 (best methods to cheat) and 4 (methods used to cheat). For research question 5, which investigates the impact of six independent variables (perception of control, goal of performance, engagement in studying, methods of cheating, peers’ influence, and institutional context) on the propensity to cheat, a hierarchical linear regression analysis with two blocks was processed. The first block was intended to control for social desirability bias (*Impression Management*) as respondents may have tried to present themselves in a socially desirable way. The second block included six predictors (independent variables) and used a stepwise procedure. SPSS (version 25) was used for the analyses.

## Results

### Research question 1: extent of cheating

The extent of the propensity to cheat on exams among respondents appears in Table 2. It shows that 15.21% of the preservice teachers agreed or strongly agreed (indicating that they have cheated repeatedly) with the statement: “I have cheated in my university studies”. Regarding their high school education, 34.90% of the preservice teachers report having cheated to increase their grades. In response to the statement “I have already glanced at my neighbor’s copy during an exam”, 27.40% of the preservice teachers either agreed or strongly agreed.

**Table 2:** Extent of cheating among respondents

	Strongly disagree		Disagree		Agree		Strongly agree	
	n	%	n	%	n	%	n	%
I have cheated in my university studies.	381	66.61	104	18.18	64	11.19	23	4.02
I have cheated in high school to increase my grades.	249	43.46	124	21.64	129	22.51	71	12.39
I have already glanced at my neighbor’s copy during an exam.	252	43.98	164	28.62	123	21.47	34	5.93

#### Research question 2: reasons for cheating

Table 3 provides the descriptive statistics for the reasons preservice teachers would cheat on exams. The most frequent reason was “if I do not think I can pass the exam” (57.59%) followed by “if the chances of getting caught are low” (43.11%) and by “if I have not studied enough” (31.06%). In contrast, the least frequent reason was “if I am running out of time” (17.63%).

**Table 3:** Reasons for cheating on exams

I will cheat if …	No		Yes	
	n	%	n	%
I do not think I can pass the exam.	243	42.41	330	57.59
The chances of getting caught are low.	326	56.89	247	43.11
I have not studied enough.	395	68.94	178	31.06
I need the highest possible mark.	434	75.74	139	24.26
My peers cheat too.	444	77.49	129	22.51
I am running out of time.	472	82.37	101	17.63

### Research question 3: best methods to cheat

As shown in Table 4, respondents consider the best methods for cheating on exams to be using notes hidden in personal belongings (63.00%) and glancing at others’ exams (55.67%). Exchanging notes with others (12.74%) and talking to peers (9.08%) received the least agreement.

**Table 4:** Best methods for cheating on exams

	No		Yes	
	n	%	n	%
Using notes hidden in personal belongings	212	37.00	361	63.00
Glancing at others’ exams	254	44.33	319	55.67
Using a cell phone or other electronic device	390	68.06	183	31.94
Hiding personal notes outside the classroom	435	75.92	138	24.08
Exchanging notes with others	500	87.26	73	12.74
Talking to peers	521	90.92	52	9.08

### Research question 4: methods used to cheat

The methods used by respondents to cheat appear in Table 5 (when measured through the *methods of cheating* factor’s items of the UCEQ). It shows that 1.92%, 2.62% and 3.85% of the preservice teachers (respectively) either agreed or strongly agreed with the statements: “I have already left the classroom during an exam to access my study notes”, “I have already used my cell phone to cheat during an exam”, and “I have already used a technological device (earpiece, tablet, etc.) to cheat during an exam”.

**Table 5:** Methods used by respondents to cheat

	Strongly disagree		Disagree		Agree		Strongly agree	
	n	%	n	%	n	%	n	%
I have already left the classroom during an exam to access my study notes.	492	85.86	70	12.22	9	1.57	2	0.35
I have already used my cell phone to cheat during an exam.	486	84.82	72	12.57	10	1.75	5	0.87
I have already used a technological device (earpiece, tablet, etc.) to cheat during an exam.	483	84.44	67	11.71	16	2.80	6	1.05

### Research question 5: impact of specific factors on the propensity to cheat

On the 573 students, 544 responded to the *Impression Management* scale and were used for the hierarchical linear regression analysis with two blocks. This analysis was conducted to examine the impact of perception of control, goal of performance, engagement in studying, methods of cheating, peers’ influence, and institutional context on the propensity to cheat.

Table 6 provides the descriptive statistics and the correlations for the seven factors and the *Impression Management* scale. Methods of cheating present the lowest mean indicating that student did not use (strongly disagree) these methods to cheat. Institutional context, engagement in studying, and propensity to cheat on exams have a mean close to disagree. This suggest that students disagree that 1) institutional context (ethical policy, code of honour, other legal frameworks) about cheating is clear; 2) they have cheated on exams; and 3) they are not engaged in studying. Perception of control and goal performance present a mean close to the middle point of the scale. This is an indication that students slightly disagree with the claims that they have no control on their preparation for exams and that they have goals of performance. Finally, students slightly agree that peers influenced the decision to cheat.

**Table 6:** Descriptive statistics and correlations (factors and IM scale)

	M	SD	2	3	4	5	6	7	8
1. Perception of control	2.34	.49	.14**	.14***	−.04	.04	.10**	.03	−.06
2. Goal of performance	2.32	.54		−.09*	.14**	.02	.09*	.05	−.03
3. Engagement in studying	2.10	.52			.09*	.12**	.15***	.18***	−.27***
4. Peer’s influence	2.61	.74				.14**	.17***	.41***	−.11**
5. Methods of cheating	1.17	.39					.08*	.45***	−.26***
6. Institutional context	1.86	.51						.03	−.06
7. Cheating on exams	1.80	.79							−.33***
8. IM scale	.39	.19							

*p ˂ .05 **p ˂ .01 ***p ˂ .001

The correlations are low (under *r* = .19) between the UCEQ factors. There are two exceptions for: propensity to cheat on exams and methods of cheating (*r* = .45) and cheating on exams and peers’ influence (*r* = .41). Engagement in studying (*r* = − 0.27), methods of cheating (*r* = −.26), and propensity to cheat on exams (*r* = −.33) present the higher correlations with *Impression Management*.

After controlling for social desirability bias (*Impression Management* scale), peer’s influence, methods of cheating, and institutional context explained 37% (Δ*R*^2^ = .37) of the variation in the propensity to cheat (see Table 7). Propensity to cheat was significantly positively associated to peers’ influence (*β =* .36) and methods of cheating (*β =* .35), and significantly negatively associated to institutional context (*β =* −.08). None of the other factors (perception of control, goal of performance, and engagement in studying) was significantly linked to the propensity to cheat. Analyses revealed no collinearity between variables as variance inflation factor was close to 1.

**Table 7:** Stepwise multiple linear regression analysis predicting decision to cheat

Predictor	Propensity to cheat	
	Δ*R*^2^	*β*
Step 1		
*Impression Management* (control variable)	.11***	−.21***
Step 2		
Peers’ influence	.15***	.36***
Methods of cheating	.11***	.35***
Institutional context	.01*	−.08*
Total *R*^2^	.37***	
n	544	

*p ˂ .05 ***p ˂ .001

## Discussion

This study examined the phenomenon of cheating on exams in faculties of education in Quebec in order to further knowledge on individual and contextual factors associated with cheating in this specific discipline. We were interested in exploring the magnitude of the phenomenon of the propensity to cheat, the reasons students put forward to justify cheating, the methods they perceived to be the best for cheating and the method used to cheat. Furthermore, the predictive value of six factors on the propensity to cheat was studied in order to pinpoint areas of focus for the reduction of cheating and the contribution to the creation of ethical organizations (Van Yperen et al. 2011).

It appears from our study that our participants do not cheat as much as is reported in the literature with numbers ranging from 30% (Williams and Williams 2012) to 52.5% (Jurdi et al. 2011). Indeed, 15.21% admitted a propensity to cheat on an exam at university. This lower percentage of students self-reporting cheating at university raises certain questions. For example, could it be that students did not want to admit to cheating at university while they were actually enrolled in a university program? This may indeed be part of the explanation since our group of students – who scored moderate on the social desirability scale – indicated that they would tend to report less cheating than what actually occurs. Another potential explanation for this low percentage could be the lack of competitiveness in teacher training programs. Student teachers do not need to have high grades to stay in their program or to find a job, especially in the current context of teacher shortages in Quebec. Once candidates are admitted in the program, they only need to pass all courses and practicums; there is no real gain associated with obtaining higher grades by cheating. In fact, when asked why they would cheat on an exam, only 24.26% of respondents indicated the pursuit of a higher mark (see Table 3) while 57.59% indicated that they would cheat if they thought it would help them to pass the exam, which in fact is the main reason to cheat evoked by our group of students. This reasoning may help explain the lower percentage of students cheating in teacher training programs. The situation is different for students in other programs such as technology, engineering, math, or business (Hensley et al. 2013) where better grades could make a difference for scholarships, internships, etc. On this question, the study conducted by Crittenden et al. (2009) is quite revealing about the high level of cheating in faculties of business worldwide.

It is interesting to note, however, that the proportion of students admitting to having a propensity to cheat during exams is more than twice as high in high school (34.90%) than in university (15.21%). This finding concurs with other studies conducted by Christensen Hughes and McCabe (2006) and Jensen et al. (2002). Furthermore, Stoesz and Los (2019), who conducted a study with secondary students in Manitoba, Canada, also found a difference in the level of cheating between younger and older secondary school students. In France, Guibert and Michaut (2009) report findings attributing more than half of the variance explaining cheating on exams to prior experiences of cheating, especially at pre-university level. To that effect, Ellahi, Mushtaq and Khan (Ellahi et al. 2013 p.660) suggest that “prior cheating experience was also positively related to rationalisation of academic dishonesty among students. It shows that prior cheating experience brings confidence and predicts future orientation of academic dishonesty among students”. Although we can appreciate the decrease in the percentage of cheaters, universities still have work to do to ensure academic integrity is respected across the board.

In our study, three factors (peers influence, methods of cheating, and institutional context) predict the propensity to cheat on exams. Two of those factors are associated with the context and one with individual. Peers’ influence is the factor that contributes most to predicting the propensity to cheat on exams. Moreover, 22.51% of our respondents selected this option as a reason for cheating on exams (Table 3). This finding is consistent with other studies indicating that peers’ cheating behavior is the best predictor of academic dishonesty (McCabe and Trevino 2002; McCabe et al. 2003) or that “there is a positive correlation between self-reported cheating and the frequencies of this behavior in mates” (David 2015 p.91). The second factor associated with the context, institutional context (ethical policy, code of honour, other legal frameworks), also presents a weak, but nonetheless significant, negative link with students’ propensity to cheat. This negative link could be expressed in such a way: students’ enhanced awareness of a university’s integrity policy and of the consequences of cheating leads to a decrease in cheating. Again, this finding concurs with previous work done by McCabe et al. (2012).

The contribution of the third factor, methods of cheating, is the second strongest in predicting the propensity to cheat on exams. Although this finding is not a surprise, since greater accessibility to various methods to cheat would facilitate student cheating, it is worthwhile exploring these results to clarify the situation. Students have clear opinions as to the best methods for cheating on exams (Table 4). The methods provided on the questionnaire fall under two categories: 1) using forbidden material (notes, electronic devices) or 2) taking, giving or receiving information from others (glancing at others’ exams, exchanging notes with others, or talking to peers). The best methods for cheating, according to respondents, are hiding notes in personal belongings (63.00%) and glancing at others’ exams (55.67%). When asked which methods they used for cheating, respondents indicated three methods out of five, although very low percentages of respondents indicated that they had used one of the methods except for glancing at others’ exams. The gap between what they see as best methods, for example using high-tech devices or cellphones to cheat (31.94%), and the usage of those methods (3.85% and 2.62%) might be explained by the fact that the examination context doesn’t allow students to have those devices with them during exams. The gap is somewhat smaller for the modality ‘glancing at others’ exams, which was considered as the best method by 55.67% of respondents and used as a method for cheating by 27.40% of respondents (see Table 2). This latter method of cheating has been identified as very popular across many cultural contexts in other studies (Bernardi et al. 2008; Dodeen 2012; Küçüktepe 2014) and authors suggest such remedies as increasing supervision during exams, having various forms of the exam by scrambling the questions on an exam, and increasing the physical distance between students (Bernardi et al. 2008). The accessibility of methods to cheat is currently enhanced with the increased popularity of online teaching and consequently, online assessments. Henceforth, efforts have to be done to adopt a variety of assessment formats, to spend time clarifying expectations in terms of students’ integrity, and to use proctoring devices. For example, assessment situations that require students to be creative, to justify their answers, or to produce elaborate answers should be used more often. As well, if using multi-choice exams is necessary, students could be asked to justify their answers and be given points for their justifications.

We were interested in exploring the impact of three factors related to students themselves, namely having goals of performance, students’ engagement in their study, and finally the perception of control they feel they have over their tasks. In our study, none of these factors are linked with the propensity to cheat on exams. Indeed, although numerous studies report a link between having performance goals and cheating (Tchouata et al. 2014; Olafson et al. 2013; Tas and Tekkaya 2010), our results rather reflect the most recent studies that suggest that the type of goals students have in mind are not predictive of the propensity to cheat (Anderman and Won 2019). As mentioned earlier, student teachers in Quebec are not in a competitive environment. Therefore, performance goals may not be as important an issue for them when compared with learning the skills and competencies they will need in their profession. We defined engagement in studying as time spent doing certain activities (going to classes, studying, etc.) and avoiding other activities (procrastinating, partying, etc.). Again, our results indicate no significant link between this factor and the propensity to cheat on exams. These results are in line with a recent study conducted by Grira and Jaeck (2019). Nor is there any correlation between the perception of control over tasks and the propensity to cheat, contrary to what other research studies are reporting (Rettinger and Kramer 2009). However, it might be the case, as reported by Rinn et al. (2014), that students’ academic skills are more closely related to the decision to cheat than their perception of control over tasks.

## Conclusion

The purpose of our study was to examine the phenomenon of cheating on exams in the faculties of education of five francophone universities in the province of Quebec, Canada. The results indicated that peers’ influence, methods of cheating, and institutional context predict the propensity to cheat on exams. Although 15.21% of the preservice teachers admitted a propensity to cheat at university, which is less than previous studies (Bens 2010; Tchouata et al. 2014), this does not mean that nothing can be done to improve the situation. Our results suggest that efforts to further reduce cheating should focus on change within the university as institution.

First, each university must have a policy on academic integrity in place and enforce it. Students must know and understand this legal framework and be aware of the consequences of cheating. McCabe and Trevino (2002) suggestion of introducing honor code to reduce cheating is also a good recommandation for institutions. However, although honors code seem to have a certain success in decreasing cheating in face-to-face assessment, Corrigan-Gibbs et al. (2015) found using stern warning to be more efficient for online assessment. Henceforth, in the current pandemic context of online assessment, a combination of approaches to prevent cheating should be put in place.

Second, among university students, specific attention should be paid to preservice teachers who will assume after graduation a dual role as leaders in professional integrity and as models of integrity for their students (Boon 2011; Cummings et al. 2007). To ensure future teachers are capable of assuming this dual role, faculties of education should initiate awareness-raising activities on cheating on exams such as discussion, case studies, role-play and practical exercises.

Third, because greater accessibility to various methods to cheat increases cheating, we also recommend intensifying the level of supervision of students during the exam sessions. Teaching assistants could help the teacher in the case of large groups. It is also possible to augment the distance between desks in an exam room, to use different versions of the same exam, and to vary the exam from year to year. These suggestions are not sufficient for online assessments for which students’ creativity and justification of anwers as well as proctoring devices are required.

Finally, to limit peers’ influence, universities should show that they value learning and integrity for all students. Preservice teachers must be made aware of the importance of genuinely developing the real-world skills that will enable them to do their jobs well.

Our study has two primary strengths: separating two phenomena – propensity to cheat on exams and plagiarism – that are often investigated as a single phenomenon, and targeting preservice teachers in faculties of education. The study also has limitations that should be taken into account when interpreting the results. One such limitation is that a self-rating method was used to collect information from a sample of 573 preservice teachers. Although we controlled for social desirability bias (*Impression Management*), we cannot guarantee beyond all doubt that the extent of propensity to cheat among respondents is as low in education faculties as we report. In addition, the results reported in this article do not allow us to explore the relation between academic skills and propensity to cheat. This exploration will be done in a future paper.

Future research could also examine how other individual characteristics of preservice teachers such as year in the study program or having to work part-time might influence cheating. It could also examine whether some interventions by universities (e.g. such as providing a three-hour lesson on cheating, stern warnings indicating the consequences of cheating during exams, etc.) would decrease the propensity to cheat.

Another avenue for future research is to understand how to promote a culture of academic integrity in universities. What kinds of activities should be done with the students? How can we better support professors so that they prevent cheating and denounce it? Answering these research questions could make a significant contribution to the training of preservice teachers who will have a crucial role to play in the education of young people in the years to come.

Finally, future research could also follow our sample of preservice teacher (or another) as they enter the teaching workforce to see whether rates of cheating in their students are correlated with teachers’ history regarding the propensity to cheat on exams.

## Data Availability

Data not available due to confidentiality of student information.

## Abbreviations

BIDR: Balanced inventory of desirable responding
CFI: Comparative fit index
CUQ: Cheating at the university questionnaire
NNFI: Non-normed fit index
RMSEA: Root mean square error of approximation
S-Bχ2: Satorra–Bentler chi-squared statistic
UCEQ: University cheating on exams questionnaire

## Footnotes

1 CFA results are SBχ^2^ = 714.69, dl = 328; NNFI = .92; CFI = .93; RMSEA = .045 [.041, 0,50]

## References

[CR1] Anderman EM, Danner F (2008). Achievement goals and academic cheating. RevInt Psychol Soc.

[CR2] Anderman EM, Midgley C (2004). Changes in self-reported academic cheating across the transition from middle school to high school. Contemp Educ Psychol.

[CR3] Anderman EM, Won S (2019). Academic cheating in disliked classes. Ethics Behav.

[CR4] Bens SL (2010). Senior education students’ understandings of academic honesty and dishonesty (doctor of philosophy).

[CR5] Bernardi RA, Baca AV, Landers KS, Witek MB (2008). Methods of cheating and deterrents to classroom cheating: an international study. Ethics Behav.

[CR6] Boon H (2011). Raising the bar: ethics education for quality teachers. Aust J Teach Educ.

[CR7] Bowers WJ (1964). Student dishonesty and its control in college.

[CR8] Callahan D (2004). The cheating culture: why more Americans are doing wrong to get ahead.

[CR9] Chaput de Saintonge DM, Pavlovic A (2004). Cheating. Med Educ.

[CR10] Christensen Hughes J, McCabe DL (2006). Understanding academic misconduct. Can J High Educ.

[CR11] Christensen Hughes J, Mighty J (2010). A call to action: barriers to pedagogical innovation and how to overcome them.

[CR12] Cizek GJ (1999). Cheating on tests: how to do it, detect it, and prevent it.

[CR13] Corrigan-Gibbs H, Gupta N, Northcutt C, Cutrell E, Thies W (2015). Deterring cheating in online environments. ACM Trans Comput Hum Interact.

[CR14] Crittenden VL, Hanna RC, Peterson RA (2009). The cheating culture: a global societal phenomenon. Bus Horiz.

[CR15] Cronan TP, McHaney R, Douglas DE, Mullins JK (2017). Changing the academic integrity climate on campus using a technology-based intervention. Ethics Behav.

[CR16] Cummings R, Harlow S, Maddux CD (2007). Moral reasoning of in-service and pre-service teachers: a review of the research. J Moral Educ.

[CR17] Cummings R, Maddux CD, Harlow S, Dyas L (2002). Academic misconduct in undergraduate teacher education students and its relationship to their principled moral reasoning. J Instr Psychol.

[CR18] D’Amours-Raymond J (2011). Balanced inventory of desirable responding.

[CR19] David LT (2015). Academic cheating in college students: relations among personal values, self-esteem and mastery. Procedia Soc Behav Sci.

[CR20] Denisova-Schmidt E, Prytula Y, Rumyantseva NL (2019). Beg, borrow, or steal: determinants of student academic misconduct in Ukrainian higher education. Policy Rev High Educ.

[CR21] Desalegn AA, Berhan A (2014). Cheating on examinations and its predictors among undergraduate students at Hawassa University College of Medicine and Health Science, Hawassa, Ethiopia. BMC Med Educ.

[CR22] Dodeen HM (2012). Undergraduate student cheating in exams. Damascus Univ J.

[CR23] Downing SM (2003). Validity: on meaningful interpretation of assessment data. Med Educ.

[CR24] Ellahi A, Mushtaq R, Bashir Khan M (2013). Multi campus investigation of academic dishonesty in higher education of Pakistan. Int J Educ Manag.

[CR25] Faucher D, Caves S (2009). Academic dishonesty: innovative cheating techniques and the detection and prevention of them. Teach Learn Nurs.

[CR26] Fendler RJ, Yates MC, Godbey JM (2018). Observing and deterring social cheating on college exams. Int J Sch Teach Learn.

[CR27] Frenette É, Fontaine S, Hébert M-H, Éthier M (2020) Étude sur la propension à tricher aux examens à l’université : élaboration et processus de validation du Questionnaire sur la tricherie aux examens à l’université (QTEU). Mesure et évaluation en éducation 42(2):1–33.

[CR28] Fishman T (2009). ‘We know it when we see it’ is not good enough: toward a standard definition of plagiarism that transcends theft, fraud, and copyright.

[CR29] Fishman T (2016) Academic integrity as an educational concept, concern, and movement in US institutions of higher learning. Handbook of academic integrity, pp 7–21

[CR30] Fontaine S, Savoie-Zajc L, Cadieux A (2013). L’impact des CAP sur le développement de la compétence des enseignants en évaluation des apprentissages. Éduc Francophonie.

[CR31] Gouvernement du Québec (2001). La formation à l'enseignement : les orientations, les compétences professionnelles.

[CR32] Grira J, Jaeck L (2019). Rationality and students’ misconduct at university: empirical evidence and policy implications. Int Educ Stud.

[CR33] Guibert P, Michaut C (2009). Les facteurs individuels et contextuels de la fraude aux examens universitaires. Rev Fr Pédagog.

[CR34] Harper R, Bretag T, Ellis C, Newton P, Rozenberg P, Saddiqui S, van Haeringen K (2019). Contract cheating: a survey of Australian university staff. Stud High Educ.

[CR35] Hensley LC, Kirkpatrick KM, Burgoon JM (2013). Relation of gender, course enrollment, and grades to distinct forms of academic dishonesty. Teach High Educ.

[CR36] Jeffrey D (2013). Profession enseignante: de la moralité exemplaire à l’éthique professionnelle. Form Prof.

[CR37] Jensen L, Arnett J, Feldman S, Cauffman E (2002). It’s wrong but everybody does it: academic dishonesty among high school and college students. Contemp Educ Psychol.

[CR38] Jurdi R, Hage HS, Chow HP (2011). Academic dishonesty in the Canadian classroom: Behaviours of a sample of university students. Can J High Educ.

[CR39] Jutras F (2013). La formation à l’éthique professionnelle:orientations et pratiques contemporaines. Form Prof.

[CR40] Kisamore JL, Stone TH, Jawahar IM (2007). Academic integrity: the relationship between individual and situational factors on misconduct contemplations. J Bus Ethics.

[CR41] Küçüktepe SE (2014). College students’ cheating behaviors. Soc Behav Personal Int J.

[CR42] Lancaster T, Clarke R (2017). Rethinking assessment by examination in the age of contract cheating.

[CR43] Ma HJ, Wan G, Lu EY (2008). Digital cheating and plagiarism in schools. Theory Pract.

[CR44] McCabe D, Butterfield KD, Trevino L (2003). Faculty and academic integrity: the influence of current honor codes and past honor code experiences. Res High Educ.

[CR45] McCabe D, Butterfield KD, Trevino L (2012) Cheating in college: why students do it and what educators can do about it. The Johns Hopkins University Press

[CR46] McCabe D, Trevino L (1993). Academic dishonesty: honor codes and other contextual influences. J High Educ.

[CR47] McCabe D, Trevino L (1997). Individual and contextual influences on academic dishonesty: a multi-campus investigation. Res High Educ.

[CR48] McCabe D, Trevino L (2002). Honesty and honor codes. Academe.

[CR49] McCabe D, Trevino L, Butterfield KD (2001). Cheating in academic institutions: a decade of research. Ethics Behav.

[CR50] Meng CL, Othman J, D’Silva JL, Omar Z (2014). Influence of neutralization attitude in academic dishonesty among undergraduates. Int Educ Stud.

[CR51] Michaut C (2013). Les nouveaux outils de la tricherie scolaire au lycée. Rech Éduc.

[CR52] Miller BL, Agnich LE, Posick C, Gould LA (2015). Cheating around the world: a cross-national analysis of principal reported cheating. J Crim Justice Educ.

[CR53] Murdock TB, Anderman EM (2006). Motivational perspectives on student cheating: toward an integrated model of academic dishonesty. Educ Psychol.

[CR54] Murdock TB, Miller AD, Goetzinger A (2007). Effects of classroom context on university students’ judgments about cheating: mediating and moderating processes. Soc Psychol Educ.

[CR55] Ndzedi F (2016). L’éthique professionnelle en enseignement : raison d’être, orientation et responsabilité. Form Prof.

[CR56] Novotney A (2011) Beat the cheat. Monit Psychol 42(6) Retrieved from http://www.apa.org/monitor/2011/06/cheat

[CR57] Olafson L, Schraw G, Nadelson L, Nadelson S, Kehrwald N (2013). Exploring the judgment-action gap: college students and academic dishonesty. Ethics Behav.

[CR58] Patrzek J, Sattler S, van Veen F, Grunschel C, Fries S (2015). Investigating the effect of academic procrastination on the frequency and variety of academic misconduct: a panel study. Stud High Educ.

[CR59] Paulhus DL (1984). Two-component models of socially desirable responding. J Pers Soc Psychol.

[CR60] Pavlin-Bernardić N, Rovan D, Pavlović J (2017). Academic cheating in mathematics classes: a motivational perspective. Ethics Behav.

[CR61] Qualls R (2014). The relationship between disciplinary practices in childhood and academic dishonesty in college students. Coll Stud J.

[CR62] Rettinger DA, Kramer Y (2009). Situational and personal causes of student cheating. Res High Educ.

[CR63] Rinn A, Boazman J, Jackson A, Barrio B (2014) Locus of control, academic self-concept, and academic dishonesty among high ability college students. J Sch Teach Learn:88–114

[CR64] Schuhmann PW, Burrus RT, Barber PD, Graham JE, Elikai MF (2013). Using the scenario method to analyze cheating behaviors. J Acad Ethics.

[CR65] Shei C (2005). Plagiarism, Chinese learners and Western convention. Taiwan J TESOL.

[CR66] Smith KJ, Derrick PL, Manakyan H (2012). A reevaluation and extension of the motivation and cheating model. Glob Perspect Account Educ.

[CR67] Stiggins RJ (2009). Assessment FOR learning in upper elementary grades. Phi Delta Kappan.

[CR68] Stiles BL, Pan M, LaBeff EE, Wong N (2017). The role of academic entitlement in college cheating: a comparison between China and the United States. Res High Educ J.

[CR69] Stoesz BM, Los R (2019). Evaluation of a tutorial designed to promote academic integrity. Can Perspect Acad Integrity.

[CR70] Sykes GM, Matza D (1957). Techniques of neutralization: a theory of delinquency. Am Sociol Rev.

[CR71] Tas Y, Tekkaya C (2010). Personal and contextual factors associated with students’ cheating in science. J Exp Educ.

[CR72] Tchouata C, Lamago MF, Singo Njabo CR (2014). Fraude aux examens et formation des enseignants : le cas de l’École normale supérieure de Yaoundé. Form Prof.

[CR73] Van Yperen NW, Hamstra MR, van der Klauw M (2011). To win, or not to lose, at any cost: the impact of achievement goals on cheating. Br J Manag.

[CR74] Walker J (2010). Measuring plagiarism: researching what students do, not what they say they do. Stud High Educ.

[CR75] Weiner B (1986). An attributional theory of motivation and emotion. An Attributional theory of achievement motivation and emotion. An Attributional theory of motivation and emotion.

[CR76] Whitley BE (1998). Factors associated with cheating among college students: a review. Res High Educ.

[CR77] Wideman M (2011). Caring or collusion? Academic dishonesty in a school of nursing. Can J High Educ.

[CR78] Williams MWM, Williams MN (2012). Academic dishonesty, self-control, and general criminality: a prospective and retrospective study of academic dishonesty in a New Zealand university. Ethics Behav.

[CR79] Yu H, Glanzer PL, Sriram R, Johnson BR, Moore B (2017). What contributes to college students’ cheating? A study of individual factors. Ethics Behav.

